# Residual beta-cell function in children with type 1 diabetes after a partial remission phase – a possible relation between C-peptide and betatrophin

**DOI:** 10.3389/fendo.2025.1602777

**Published:** 2025-06-18

**Authors:** Emilia Odyjewska, Monika Kupińska, Milena Jamiołkowska-Sztabkowska, Artur Bossowski, Barbara Głowińska-Olszewska

**Affiliations:** Department of Pediatrics, Endocrinology, and Diabetology with Cardiology Division, Medical University of Bialystok, Białystok, Poland

**Keywords:** T1D (Type 1 diabetes), children, C-peptide, obesity, betatrophin, islet beta (β) cells

## Abstract

**Introduction:**

Maintaining endogenous insulin secretion in type 1 diabetes (T1D) long after its onset, and thus the need for early diagnosis and searching for factors preserving the secretory function of β-cells, has become an important goal of current research. The aim of the study was to evaluate C-peptide secretion in T1D children with at least 1 year disease duration and to investigate the potential role of body mass index (BMI) and betatrophin on residual β-cell function. We also assessed factors that may affect betatrophin levels.

**Methods:**

121 children and adolescents suffering from T1D were divided into groups based on: clinical significance of C-peptide; BMI-SDS <1 and ≥1; and disease duration to compare C-peptide and betatrophin levels and determine the importance of these changes.

**Results:**

Of the children recruited, 44 (36.36%) had clinically significant C-peptide (> 0.23 ng/ml), and compared with the group with clinically insignificant C-peptide they had later onset (P<.001), shorter duration of illness (P<.001), lower daily insulin requirement (P=.025), lower mean HbA1c over the past year (P=.002), higher betatrophin levels (P=.019), and BMI-SDS at diagnosis (P=.013). Betatrophin levels correlated positively with C-peptide (P=.043) while negatively with patient’s age (P<.001), BMI-SDS (P=.010), disease duration (P=.006), HbA1c level at sampling (P=.022), average HbA1c level over the past year (P=.006), and basal insulin (P=.001).

**Conclusion:**

The positive significant relationship between betatrophin and C-peptide concentrations may indicate betatrophin as a potential biomarker of long-lasting residual β-cell function. Negative correlation with BMI identifies the ongoing need to maintain an appropriate body mass.

## 1 Introduction

Type 1 diabetes (T1D) is a chronic metabolic disease characterized by autoimmune destruction of pancreatic β-cells, leading to endogenous insulin deficiency. At present, the basic method of treating this disorder is exogenous supplementation of insulin with hormone analogues through multiple daily injections (MDI) or continuous subcutaneous insulin infusion (CSII). Unfortunately, current treatment methods for children with diabetes, despite the continuous improvement of glucose control systems such as continuous glucose monitoring (CGM) or closed-loop insulin pumps, are not always sufficient to maintain normoglycemia. Preservation of residual β-cell function has been shown to be associated with better glycemic control and has a protective effect on both acute and long-term complications of diabetes. This prompts the search for factors that enhance β-cell function, or at least maintain their longer survival as well as the search for biomarkers of the remission phase. The discovery of methods and factors to expand the secretory function of pancreatic islets would provide new therapeutic options that would not only improve metabolic parameters, but also have a positive impact on patients’ quality of life (1).

The biomarker commonly used to assess pancreatic β-cell function is C-peptide. It is generated from the processing of preproinsulin molecules into insulin and C-peptide and abnormalities in this process, have clinical relevance to T1D (2). Shortly after initiating treatment, partial remission is usually observed (3). This period is characterized by preserved C-peptide secretion and better glycemic control with lower daily insulin requirements (DIR). According to International Society for Pediatric and Adolescent Diabetes (ISPAD), remission criteria include HBA1c <7% with DIR <0.5 U/kg/24h (4). However, after the relapse, C-peptide level begins to drop dramatically and is usually undetectable after the clinical onset (5). Nonetheless, it is already proved that C-peptide can remain at residual levels in some patients for years (6). The Diabetes Control and Complications Trial (DCCT) indicates that sustained C-peptide concentrations are associated with lower rates of hypoglycemia, lower HbA1c levels, reduced insulin dose requirements and lower incidents of diabetes complications, such as retinopathy and nephropathy (7). These findings suggest that even patients with long-standing and advanced disease may benefit from interventions to protect β-cell function or to prevent complications.

Numerous studies have been conducted to find factors influencing the presence and duration of partial remission phase (5, 8, 9). Unfortunately, little is known about the factors that predispose for a preservation of C-peptide, when partial remission phase ends. In recent years, several new candidate biomarker of residual β-cell function have emerged. One of the potentially significant factors could be betatrophin, a hormone alternatively referred to as angiopoietin-like protein 8 (ANGPTL8), hepatocellular carcinoma-associated protein (TD26), or lipazin, which is synthesized primarily in the liver and moderately in adipose tissue, adrenal glands, duodenum, and small intestine (10). Betatrophin has been studied mainly in patients with early T1D, but its elevated blood levels have also been observed in adults with long-term diabetes (11). However, little research has been conducted on this topic in children. Studies on mice have shown a significant effect of betatrophin in serum on the increase in triglyceride levels and a positive effect on the proliferation of pancreatic β-cells (12, 13). Betatrophin has been found to induce positive activators of Ki67 protein signaling and proliferation, such as cyclin A1, cyclin F, and E2F2, and inhibit suppressors cdkn1a and cdkn2a in pancreatic β-cells. Furthermore, overexpression of betatrophin levels improves glucose tolerance and lowers fasting blood glucose levels (14). In the presence of high glucose concentrations, insulin induces an increase in betatrophin levels, which activates β-cells, their proliferation, and stimulates them to release insulin, take up glucose, and enter the cells. Once glycemia is normalized, the positive feedback is stopped, and insulin-induced betatrophin production is inhibited (14).

Studies on human population explaining the role of betatrophin in glucose metabolism and in insulin resistance are few and have not yet fully clarify the issue. It has been found that betatrophin levels are increased in children with T1D compared to their healthy peers, particularly at the onset of the disease, when betatrophin levels may increase up to threefold, and high serum concentrations persist in the early stages of the disease. The downward trend in serum betatrophin concentrations starts after approximately one year (5), which may be related to the exploitation of pancreatic islets with the duration of the disease. Studies conducted on Asian Indians showed that patients with excess body weight (BMI > 25 kg/m2) had lower blood betatrophin levels than normal weight patients, regardless of whether they were type 2 diabetics (T2D) or patients with normal glucose tolerance. Furthermore, betatrophin showed a strong negative correlation with waist circumference, insulin resistance, fasting and 2-hour postprandial glucose, and HbA1c, while it showed a positive correlation with fasting C-peptide (15). It was hypothesized that the increased betatrophin concentration in patients suffering from diabetes was not due to insulin deficiency but to insulin resistance (11). When a patient with T1D has genetic susceptibility factors and contributing environmental factors such as obesity, chronic inflammation, hormonal changes, or a long duration of the disease, a condition called double diabetes may occur, in which the clinical features of both types of diabetes overlap (16).

Many publications have shown C-peptide secretion during remission in children with diabetes, but scarcely any studies have addressed the period after remission and several years of disease in this population, when C-peptide may continue to be secreted. In our previous work, we indicated that above 30% of children with long-standing diabetes have clinically significant C-peptide levels (>0.23 ng/ml according to DCCT recommendations) and we confirmed that it involves better metabolic control in these patients (17).

The aim of the current study was to assess residual β-cell function in children with type 1 diabetes, with at least 1 year of disease duration, who had exited remission phase and to investigate the significance of BMI and its effect on C-peptide secretion. Additionally, we decided to test the hypothesis, that there would be a difference in betatrophin concentration between individuals with and without clinically significant C-peptide level. Subsequently, we aimed to assess the correlation between circulating betatrophin and clinical factors that may affect its levels. We decided not to include patients with disease duration of less than 1 year in our analysis because the patients’ condition was not yet stabilized, treatment decisions (CSII or MDI) were still pending, and they had significantly higher betatrophin levels at that time, which may have confounded our analyses.

## 2 Materials and methods

Our retrospective study, involving data collected between January 2020 and December 2022, included 121 children and adolescents aged 3–18 years (12.98 ± 3.54 years), patients of the Diabetes Outpatient Clinic for Children of the University Children’s Clinical Hospital in Bialystok, Poland, who were diagnosed with type 1 diabetes according to ISPAD criteria (18), with the disease duration of at least 1 year and not meeting the criteria for partial remission according to ISPAD: HBA1c <7% with DIR < 0.5 U/kg/24h (4). Exclusion criteria were the diagnosis of diabetes other than type 1, age over 18 years and the duration of the disease less than 1 year and meeting the remission phase criteria.

The data of 121 patients (61 boys, 60 girls) were analyzed. The diabetes duration ranged from 1 to 15 years (5.47 ± 3.89 years). All children were treated with intensive insulin therapy: 26 (21.49%) - with MDI, 95 (78.51%) - treated with CSII using an insulin pump. Anthropometric data have been assessed. Height and weight were measured using a medical scale and an anthropometer in the morning in light clothing. Body weight was measured to the nearest 100 g, and height to the nearest 0.5 cm. BMI was calculated by dividing children’s weight in kilograms by their height in meters squared. To adjust for age and gender, the BMI-SDS was calculated using sex-specific and age-specific BMI growth charts based on Polish OLAF study (19). BMI-SDS < 1 indicates normal body weight, while BMI-SDS ≥ 1 signifies overweight and obesity according to WHO (20).

A blood sample (2.7 cm per clot) was taken to perform the test. C-peptide level was used as an indicator of the maintenance of endocrine pancreatic function due to its secretion in equimolar proportions with insulin. The concentration of fasting C-peptide was determined using the traditional method, as well as the modern ultrasensitive C-peptide ELISA method (Mercodia, Sylveniusgatanm, Sweden) using ELx 800 Automated Microplate Reader, Bio-Tek Instruments, Vermont, USA with a lower detection limit of < 2.5 pmol/L, which corresponds to 0.0076 ng/ml. Clinically significant fasting C-peptide levels according to DCCT were established at > 0.23 ng/ml (7). Betatrophin concentration was assessed using the ELISA method (BioVendor, Brno, Czech Republic) which was specific for human betatrophin and had a sensitivity of 0.244 ng/ml. HbA1c levels were determined using monoclonal antibodies in a biochemical analyzer (Co bas, Integra 800, Roche, Switzerland). Additionally, an analysis of clinical data was performed to assess the factors influencing the maintenance of residual C-peptide, hence endogenous insulin secretion: patient’s age, disease duration, age of onset, duration of clinical remission, DIR, HbA1c level at diagnosis, at collection and average value over the last year (average of three or four measurements taken every three months), BMI-SDS at disease onset, and actual BMI-SDS.

For the purpose of our analysis and comparison of various parameters, patients were categorized into groups based on the secretion of clinically significant (N=44) and insignificant (N=77) C-peptide levels; BMI-SDS < 1 (N=85) and ≥ 1 (N=36); and into 3 groups according to the duration of the disease: the first - up to 2 years (N=35), the second - 2–5 years (N=34) and the third - more than 5 years (N=52).

## 3 Statistical analysis

Statistical analysis was performed using Statistica version 13.3 (StatSoft Krakow, Poland). The Kolmogorov-Smirnov test of normality with Lillefors correction and Shapiro-Wilk test were used to determine whether all continuous variables have a normal distribution. All examined variables with a parametric distribution were evaluated using the unpaired Student’s T-test to compare the differences between the groups. As several of the study parameters were not normally distributed, the two-tailed Mann-Whitney U-test was used to correlate continuous variables. To assess the relationship between two qualitative variables, Pearson correlation was used for parametric data and Spearman rank coefficient for nonparametric data. Multiple linear regression analysis was performed to detect independent determinants of C-peptide levels. All data are expressed as mean ± SD or median with interquartile range (IQR), as appropriate. Relative risk was evaluated with the use of Fishers’exact test, and calculation of 95% confidence interval with Koopman asymptomatic score. For Student’s T-test, with α=.05, assumed medium effect size (0.5) and our population size, estimated power of the test was calculated as.803-.837 depending on the subgroup sizes. Subsequently, for correlation analysis, with our sample size, α=.05 and medium effect size (0.3), estimated power of the test reaches up to.95. For linear multiple regression with six predictors, our sample size, α=.05 and assumed medium effect size (0.15) the calculated power was estimated at.895.

Differences were considered statistically significant with P-value <.05.

## 4 Ethical approval

The study protocol was approved by the Ethical Committee of the Medical University of Bialystok (R-I-002/402/2019). Written informed consent was obtained from parents/legal guardians as well as from patients aged > 16 years.

## 5 Results

The study covered 121 Caucasian children, with almost equal participation of both sexes (50.41% boys). Clinical and biochemical characteristics of the study group were collected in **Table 1**.

**Table 1:** Clinical and biochemical characteristics of all study patients and comparison of groups with clinically significant and insignificant fasting residual C-peptide.

Parameter	All patients	Clinically SIGNIFICANT fasting residual C-peptide	Clinically INSIGNIFICANT fasting residual C-peptide	P-value
No. of patients	121	44	77	–
Male, %	50.41%	61.36%	44.16%	–
Age [years]	12.98 ± 3.54	12.96 ± 3.25	13.00 ± 3.72	.803
Age at onset [years]	7.53 ± 3.94	10.28 ± 3.02	5.96 ± 3.53	.000
Disease duration [years]	4.50 (2.00 - 8.00)	2.00 (1.5 – 3.00)	6.00 (4.00 - 10.00)	.000
Remission time [months]	8.00 (0.00 - 12.00)	10.00 (7.00 - 13.00)	4.00 (0.00 - 10.00)	.000
BMI [kg/m2] at onset	16.26 ± 2.91	17.34 ± 3.06	15.54 ± 2.59	.001
BMI-SDS at onset	-0.22 ± 1.03	0.07 ± 0.96	-0.41 ± 1.04	.013
BMI [kg/m2] (actual)	20.16 ± 3.60	19.81 ± 3.33	20.36 ± 3.76	.549
BMI-SDS (actual)	0.59 ± 1.05	0.43 ± 0.88	0.68 ± 1.13	.346
Betatrophin [ng/ml]	6.595 (3.445 - 12.345)	8.318 (4.288 - 21.478)	5.850 (3.345 – 9.710)	.016
Fasting C-peptide [mg/dl]	0.040 (0.010 - 0.270)	0.390 (0.165 – 0.645)	0.010 (0.010 - 0.030)	.000
Fasting C-peptide [ng/mL] (ultrasensitive method)	0.124 (0.007 - 0.656)	1.641 (0.470 - 2.626)	0.008 (0.008 - 0.105)	.000
HbA1c at onset [%]	10.40 ± 2.35	11.33 ± 2.49	9.85 ± 2.09	.001
HbA1c at sample collection [%]	7.50 (7.02 - 8.45)	7.44 (6.99 - 8.25)	7.60 (7.05 - 8.68)	.279
average HbA1c over the last year [%]	7.37 (6.89 - 7.94)	7.10 (6.71 -7.44)	7.56 (7.05 - 8.33)	.002
DIR [U/kg/24h]	0.755 ± 0.187	0.707 ± 0.187	0.782 ± 0.183	.025
Total cholesterol [mg/dl]	168.35 ± 28.08	165.82 ± 32.58	169.79 ± 25.28	.329
HDL- C [mg/dl]	66.72 ± 17.63	64.36 ± 17.90	68.06 ± 17.45	.170
LDL- C [mg/dl]	94.05 ± 24.77	94.34 ± 28.88	93.88 ± 22.30	.824
Triglycerides [mg/dl]	63.00 (51.00 - 80.00)	63.00 (47.00 - 80.00)	63.08 (51.00 - 80.00)	.586

Results are presented as mean ± SD or median with quartile range. P values <.05 are in bold.

BMI, body mass index; BMI-SDS, BMI SD score; DIR, daily insulin requirement; HDL-C, high-density lipoprotein cholesterol; LDL-C, low-density lipoprotein cholesterol.

The ultrasensitive method showed detectable levels of C-peptide in 54 (44.63%) individuals, with the lowest detection limit of 0.0076 ng/ml found. Clinically significant C-peptide level (> 0.23 ng/ml) was found in 44 (36.36%) patients. The mean age of children in both groups (clinically significant *vs*. insignificant C-peptide) was comparable, but patients with higher C-peptide levels were older at diagnosis (10.28 ± 3.02 *vs*. 5.96 ± 3.53 years, P <.001). As might be expected, children with preserved clinically significant C-peptide had a shorter disease duration (P <.001) and a longer mean remission time (P <.001). The BMI-SDS value at the beginning of the disease was higher in the group of patients with significant C-peptide level than in the remaining group of patients (0.07 ± 0.96 *vs*. -0.41 ± 1.04, P = .013), yet no such difference was found in the current BMI-SDS (P = .346). We also found statistically significant lower average HbA1c from the last year (P = .002) as well as lower DIR (P = .025) and higher betatrophin concentrations (P = .016) in patients with clinically significant compared to subjects with clinically insignificant residual fasting C-peptide levels (**Table 1**).

**Figure 1:**
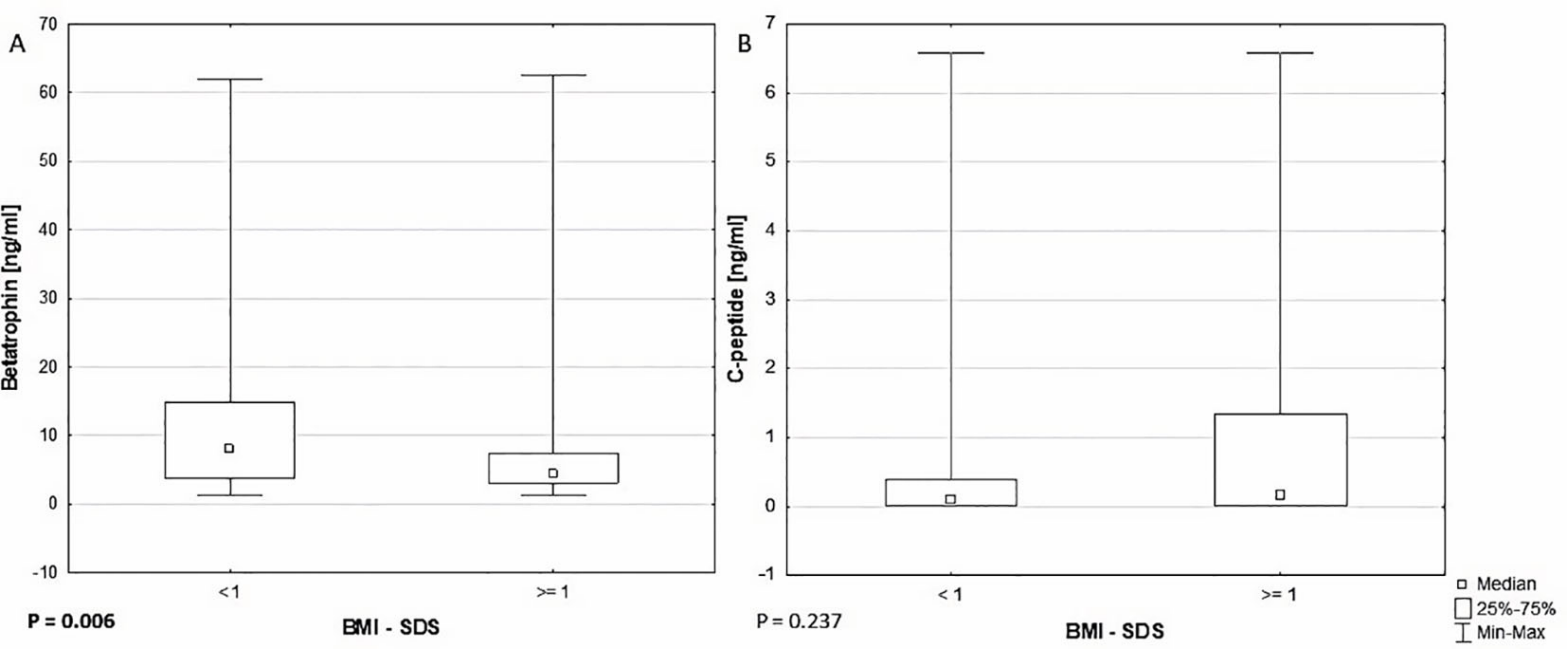
Comparison of **(A)** betatrophin (P = .006) and **(B)** C-peptide (P = .237) levels between BMI-SDS <1 and ≥1 groups.

There were noticeable differences between the groups of patients with BMI-SDS <1 and ≥1 in betatrophin concentrations 7.970 (3.790 - 14.780) *vs*. 4.332 (3.110 - 7.470) ng/ml, P = .006 (**Table 2**, **Figure 1A**). Patients with BMI-SDS <1 were younger (12.50 ± 3.34 *vs*. 14.12 ± 3.79, P = .007) had earlier disease onset (7.05 ± 3.56 *vs*. 8.67 ± 4.57, P = .039) and surprisingly, had higher DIR (0.783 ± 0.194 *vs*. 0.686 ± 0.152, P = 0.016) than patients with greater body weight. The analysis did not reveal any statistically significant differences in C-peptide levels between these groups (P = .237) (**Figure 1B**). It should be added that already at the time of diagnosis there was a significant difference in BMI and BMI-SDS values between these groups (both P <.001), which could have affected both C-peptide and betatrophin levels from the very beginning of the disease.

**Table 2:** Comparison of clinical and biochemical features between BMI-SDS <1 and ≥1 groups.

Parameter	BMI-SDS <1	BMI-SDS ≥1	P-value
No. of patients	85	36	–
Male, %	52.94%	44.44%	–
Age [years]	12.50 ± 3.34	14.12 ± 3.79	.007
Age at onset [years]	7.05 ± 3.56	8.67 ± 4.57	.039
Disease duration [years]	4.50 (2.50 - 8.00)	4.75 (1.75 - 7.75)	.635
Remission time [months]	8.00 (0.00 - 12.00)	8.50 (0.00 - 12.50)	.695
BMI [kg/m2] at onset	15.16 ± 2.23	18.82 ± 2.71	.000
BMI-SDS at onset	-0.55 ± 0.94	0.58 ± 0.78	.000
BMI [kg/m2]	18.65 ± 2.32	23.73 ± 3.60	.000
BMI-SDS	0.12 ± 0.53	1.71 ± 1.12	.000
Betatrophin [ng/ml]	7.970 (3.790 - 14.780)	4.333 (3.110 - 7.470)	.006
Fasting C-peptide [mg/dl]	0.020 (0.010- 0.170)	0.080 (0.010- 0.345)	.237
Fasting C-peptide [ng/mL] (ultrasensitive method)	0.086 (0.080 - 0.395)	0.165 (0.008 - 1.345)	.237
HbA1c at onset [%]	10.33 ± 2.43	10.57 ± 2.15	.606
HbA1c at sample collection [%]	7.50 (7.00 - 8.27)	7.53 (7.11 - 8.63)	.596
average HbA1c over the last year [%]	7.30 (6.83 - 7.90)	7.41 (7.08 - 8.12)	.318
DIR [U/kg/24h]	0.783 ± 0.194	0.686 ± 0.152	.016
Total cholesterol [mg/dl]	167.61 ± 27.43	170.08 ± 29.90	.858
HDL- C [mg/dl]	68.04 ± 17.28	63.61 ± 18.29	.192
LDL- C [mg/dl]	91.94 ± 23.77	99.03 ± 26.69	.140
Triglycerides [mg/dl]	68.55 ± 25.98	75.94 ± 46.05	.896

Results are presented as mean ± SD or median with quartile range. P values <.05 are in bold.

BMI, body mass index; BMI-SDS, BMI SD score; DIR, daily insulin requirement; HDL-C, high-density lipoprotein cholesterol; LDL-C, low-density lipoprotein cholesterol.

**Figure 2:**
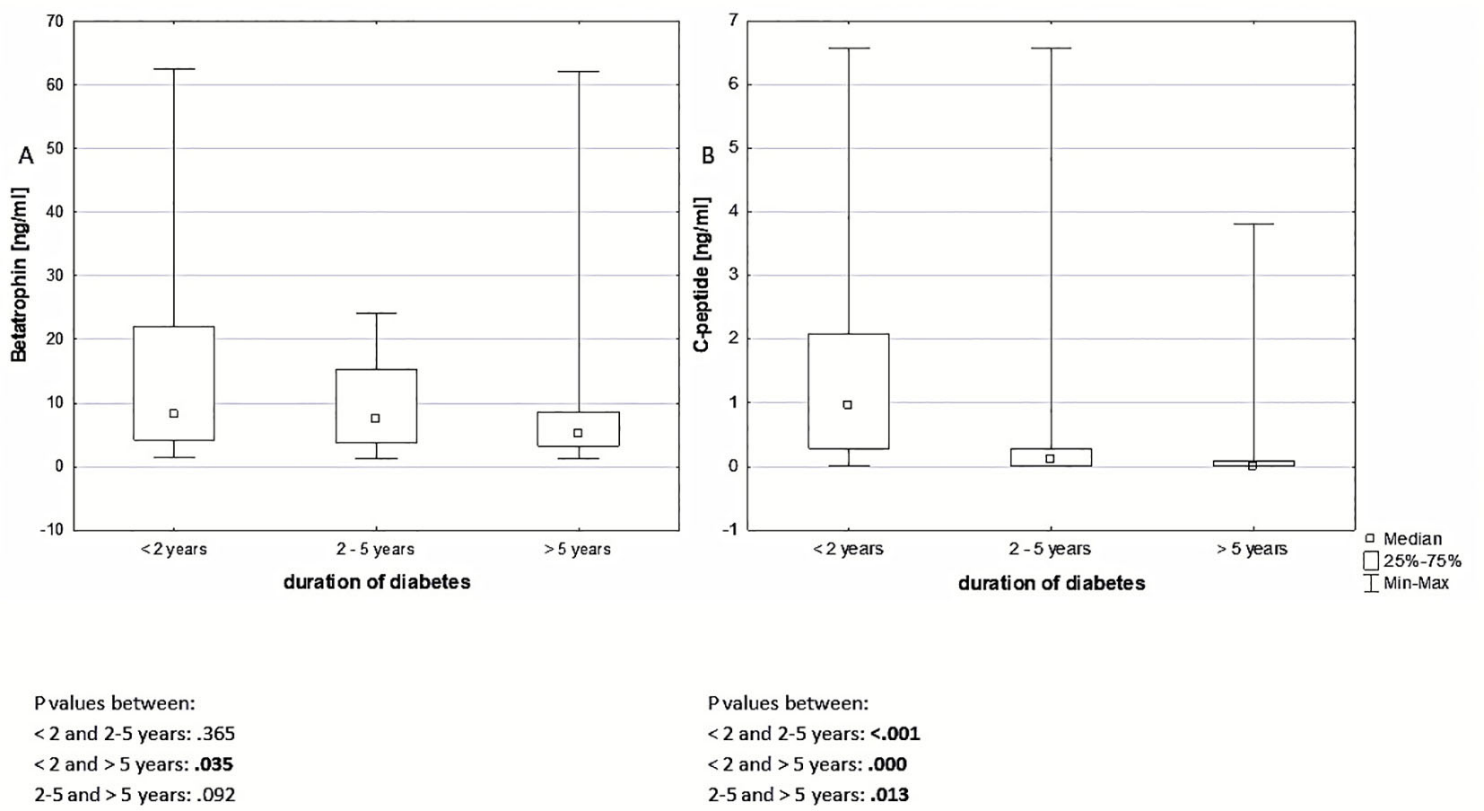
Differences in betatrophin **(A)** and C-peptide **(B)** concentrations among groups with disease duration < 2 years, 2-5years and > 5 years. P values <.05 are in bold.

In the subgroups according to the duration of diabetes, statistically significant difference in betatrophin levels was observed exclusively between the groups with disease duration up to 2 years and greater than 5 years (P = .035) (**Figure 2A**). It should be noted, however, that children with diabetes of a shorter duration were usually younger (11.71 ± 3.47 *vs*. 14.38 ± 3.04 years old, P <.001), diagnosed at a later age (often during puberty) (10.14 ± 3.50 *vs*. 5.36 ± 3.30 years old, P <.001), and had lower DIR (.672 ± .155 *vs*.796 ± .179 U/kg/24h, P = .003). C-peptide values showed a decreasing trend with disease duration: in the group with disease duration <2 years (0.966 (0.288 - 2.087) ng/ml), 2–5 years (0.118 (.008 - 0.288) ng/ml), and >5 years (0.008 (0.008 - 0.095) ng/ml) (**Figure 2B**).

**Figure 3:**
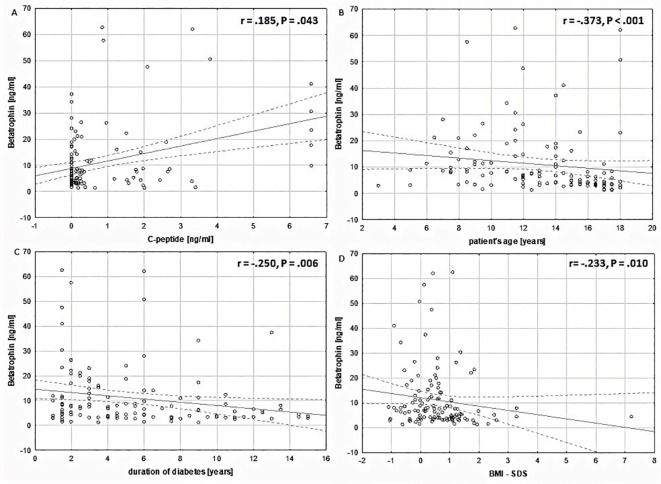
Correlation analysis of plasma betatrophin concentrations (ng/ml) in relation to the studied variables within the entire study group. **(A)** in relation to C-peptide levels (ng/ml) (Spearman r = .185, P = .043). **(B)** in relation to patient’s age (Spearman r = -.373, P <.001). **(C)** in relation to duration of diabetes (Spearman r = -.250, P = .006). **(D)** in relation to BMI-SDS (Spearman r= -.233, P = .010).

Interestingly, the results of the statistical analysis showed a positive correlation between the levels of betatrophin and C-peptide (R = 0.185, P = 0.043). It was found that the level of betatrophin is negatively correlated with the patient’s body weight (R = -0.383, P <.001) as well as with BMI (R = -0.385, P <.001) and BMI-SDS (R = -.0233, P = .010), the duration of the disease (R = -0.249, P = 0.006), patient’s age (R = -0.373, P <0.001), HbA1c at sample collection (R = -0.208, P = 0.022), average HbA1c over the last year (R = -0.248, P = 0.006), and basal insulin (R = -0.314, P = .001) (**Table 3**, **Figures 3A–D**). No correlation was found between the level of betatrophin and the level of triglycerides (TG), total cholesterol (TC), neither high density lipoprotein cholesterol (HDL-C) nor low density lipoprotein cholesterol (LDL-C) (respectively: P = 0.903; P = 0.468; P = 0.921; P = 0.667).

**Table 3:** Correlation analysis between betatrophin concentrations and studied variables within the entire study group.

Parameter	Betatrophin concentration
Age	**r =** -.**373; p <.001**
Age at onset	r = -.021; p = .816
Disease duration	**r =** -.**250; p = .006**
BMI	**r =** -.**385; p <.001**
BMI-SDS	**r =** -.**233; p = .010**
Fasting C-peptide level	**r = .185; p = .043**
HbA1c at onset	**r = .236; p = .016**
HbA1c at sample collection	**r =** -.**208; p = .022**
avg. HbA1c over the last year	**r =** -.**247; p = .006**
DIR	r = .116; p = .206
Total cholesterol	r = .066; p = .468
HDL- C	r = .009; p = .921
LDL- C	r = .039; p = .667
Triglycerides	r = -.011; p = .073

P values <.05 are in bold.

BMI, body mass index; BMI-SDS, BMI SD score; avg, average; DIR, daily insulin requirement; HDL-C, high-density lipoprotein cholesterol; LDL-C, low-density lipoprotein cholesterol.

Regression analysis was performed and showed that greater betatrophin levels (β=.263, P=.003), older age at disease onset (β=.316, P=.004) and undeniably duration of remission (β=.194, P=.035) were factors determining higher concentrations of preserved C-peptide (**Table 4**).

**Table 4:** Linear regression of variables independently affecting C-peptide levels in youths with type 1 diabetes at least one year after diagnosis.

Parameter	Unstandardized coefficients		Standardized coefficient		
	*B*	*SE*	β	*T*	*p*
F (6,90)=10.22; p <.001; R^2adj.^=.365 (Constant)	-0.035	1.016		-0.035	.972
Betatrophin [ng/ml]	0.031	0.010	0.263	3.060	.003
Age at onset [years]	0.125	0.042	0.316	2.944	.004
Disease duration [years]	-0.038	0.040	-0.094	-0.931	.355
Remission time [months]	0.038	0.018	0.194	2.145	.035
Daily insulin requirement [U/kg/24h]	-0.945	0.717	-0.116	-1.318	.191
HbA1c at onset [%]	0.014	0.061	0.022	0.233	.816

Dependent variable: C-peptide [ng/ml]. P values <.05 are in bold.

## 6 Discussion

The aim of our study was to determine whether children suffering from type 1 diabetes for at least 1 year, beyond the remission phase can still secrete C-peptide in clinically significant amounts and factors that may affect its levels. Among the recruited children, 44 (36.36%) had detectable, clinically significant levels of C-peptide. We also aimed to assess the potential role of betatrophin as a biomarker of residual β-cell function. We reported that betatrophin levels may be influenced by body weight, age or duration of the disease. To our knowledge, for the first time we present a positive correlation between the concentration of betatrophin and C-peptide in the discussed population, with betatrophin being one of the strongest predictors among the factors correlating with C-peptide.

The study by Gokulakrishnan et al. did show a positive correlation between betatrophin and C-peptide, but included a population with T2D (15). Another study showed an increase in both betatrophin and C-peptide in patients with T2D, but no clear relationship between these two parameters was observed. However, the same study showed a positive correlation between betatrophin and C-peptide in non-diabetics (21). C-peptide levels are thought to reflect residual insulin reserves (22). Studies confirm the correlation between detectable C-peptide levels in long-term diabetes and good metabolic control, and additionally, maintained residual pancreatic function seems to play an important role in preventing serious complications such as hypoglycemia, nephropathy or retinopathy (23). The interdependence between betatrophin and C-peptide may indicate a positive effect of betatrophin on pancreatic secretion as well as on remaining in the partial remission phase. As with C-peptide levels, betatrophin levels decrease with longer disease duration, but not as rapidly. In addition, we found that betatrophin levels were negatively correlated with HbA1c at the time of sample collection and the average HbA1c level over the past year. This findings is consistent with the notion that higher betatrophin levels may contribute to better glycemic control in the pediatric T1D population. On the contrary, the study by Espes et al. suggests that although betatrophin levels are increased in patients with type 1 diabetes, its levels do not correlate with C-peptide levels, glycemic control, or insulin requirements (11).

Additionally, obesity appears to have an impact on betatrophin levels, but there are discrepancies in the results. The first studies did not show a significant changes in ANGPTL8 between obese and normal weight participants (24, 25). A little later, a study was published presenting higher levels of ANGPTL8 among obese people compared to a lean control group (26). Increased levels of betatrophin were observed in obese teenagers compared to subjects with normal body weight, regardless of gender (27). In another study, a statistically significant positive correlation between BMI and betatrophin levels in patients, in general, in partial remission, and 1.5 years after the diagnosis of T1D was observed, but there was no correlation at the onset of the disease (5). Conversely, circulating betatrophin levels were significantly reduced in people with obesity and reduced even more in participants with impaired glucose tolerance and T2D. They were also found to be positively correlated with a quantitative index of insulin sensitivity and HDL-C levels (28). Tuhan et al. have also reported reduced betatrophin levels in obese patients as well as those with insulin resistance in the pediatric population (29). Our findings regarding the correlation between the metabolic parameter both BMI and BMI-SDS and the level of circulating betatrophin are consistent with the results of the latter. Moreover, as indicated by numerous scientific reports, obesity may cause a state of chronic inflammation, which may affect the functioning of the organs producing betatrophin, among others the liver, and thus alters its concentrations (30). It can be assumed that obesity, coexisting with T1D, and its dysregulatory effect on the body may reduce the secretion of betatrophin and accelerate the loss of pancreatic β-cells, manifested by a faster decrease in C-peptide (31), which could imply that maintaining a healthy body weight may contribute to prolonging the residual function of pancreatic cells. Nevertheless, studies have also raised the issue of a negative correlation between betatrophin and insulin resistance, which may suggest that not the increased body weight itself, but the insulin resistance present in obesity may have an impact on the decrease in betatrophin concentration (29, 32).

Most previous studies reported increased levels of betatrophin at the onset of the disease. Similarly to the others we detected high values of betatrophin concentrations in children up to the second year of the disease, however, the longer the duration of diabetes, the concentrations decrease. Villalba et al. showed increased betatrophin concentrations in children and adolescents with T1D up to three times at the time of diagnosis when compared to control subjects. They also found a tendency for betatrophin levels to decrease from the twelfth month of the disease (P = .057) (5). Increased levels of betatrophin were also observed in adolescent Saudi females diagnosed with T1D, compared to their healthy peers (33). Another analysis also found elevated levels of circulating betatrophin, this time in a population of adults with T1D (11). However, the influence of disease duration on betatrophin concentration has not been studied. Studies have shown that betatrophin levels are also elevated in T2D patients and may be influenced by the duration of the disease (25, 34–36). This suggests that hyperglycemia may play a key role in increasing betatrophin levels in diabetes at least at the beginning of the disease. It is alluring to speculate that increased betatrophin concentrations may have had a temporary positive effect on β-cell stimulation at the onset of the disease or that the lack of betatrophin causes excessive reduction of residual C-peptide. However, in the long term, it does not protect against the progressive loss of residual C-peptide. Nevertheless, in-depth researches on the mechanism of action of betatrophin may enable treatment with this molecule in the future.

In contrast to other studies, we did not obtain any significant correlation between betatrophin and the lipid profile in the analyzed group. Ghasemi et al. found that in diabetic patients the level of betatrophin was positively correlated with the levels of TG and TC with statistical significance (37). Circulating betatrophin concentrations have been shown to be significantly lower in patients with dyslipidemia characterized by high TG levels or low HDL-C levels (38). A cross-sectional study conducted in a Chinese population presented that circulating concentrations of full-length betatrophin were significantly lower in patients without dyslipidemia compared with patients with documented dyslipidemia. The mentioned betatrophin concentrations were positively correlated with the levels of non-HDL-C, TG and TC, and negatively with the concentration of HDL-C (39). In another study, circulating ANGPTL8 concentrations were an important factor positively associated with fasting hypertriglyceridemia in diabetic patients, while negatively correlated with levels of HDL-C (40).

More recently, betatrophin has been extensively studied for its ability to promote β-cell proliferation, nevertheless the cause and significance of increased betatrophin levels in type 1 diabetes remain undetermined. Its correlation with metabolic and endocrine diseases such as diabetes or obesity is of great interest. Many studies have been conducted in the pediatric population in remission of T1D, as well as in long-lasting disease. We decided to focus on a relatively understudied group, i.e. differences between children fresh out of clinical remission compared to those with longer duration of disease in the search for still secreting C-peptide, in the context of the importance of betatrophin as a potential marker of preserved residual beta-cell function and its differences between overweight and normal-weight children. One of the possible implications of our findings for future research is that stimulating pancreatic β-cells proliferation through betatrophin to prolong insulin or residual C-peptide secretion to maintain blood glucose levels within desired limits in T1D patients would be a beneficial mechanism, but its presence remains to be determined.

### 6.1 Limitations

Limitations of this study include its observational design and the lack of measurement of some variables. Therefore, we cannot analyze changes between ANGPTL8 levels at diagnosis and at sampling in relation to, for example, C-peptide or changes in HbA1c, nor the rate of decline in individual patients due to the limited number of events. Due to the retrospective nature of the above study, the CGM results, i.e. time in range (TIR), time above range (TAR), time below range (TBR), glucose management indicator (GMI) or coefficient of variability (CV), which are increasingly appearing in newer publications on T1D, are not available and we cannot refer to betatrophin concentration in terms of short-term glycemia. However, we believe that the significance of transient glucose fluctuations is not as important for fluctuations in the concentration of the studied molecule as persistent metabolic control, which is reflected by HbA1c.

## 7 Conclusion

A large part of the pediatric type 1 diabetes population can still secrete clinically significant amounts of C-peptide even in the presence of long-term disease, which may promote better metabolic control. Therefore, it is important to search for methods and factors that influence the extension of pancreatic secretory function- betatrophin may be one of the possibilities. Recent studies indicate the presence of elevated betatrophin levels in people with type 1 diabetes. The most important finding of our study is that circulating betatrophin levels correlate positively with C-peptide, which, combined with the negative association of betatrophin with HbA1c, may suggest that betatrophin contributes to better glycemic control. The main clinical implication of our results is the potential use of betatrophin as a future biomarker of residual β-cell function. Additionally, excess body weight appears to have a negative impact on betatrophin levels in children with T1D, thus maintaining a healthy body weight may protect against betatrophin deprivation, which may lead to improved glycemic outcomes. Many variables, such as nutritional status, duration of the disease, lipid or metabolic profile, may influence changes in both C-peptide and betatrophin concentrations. At this time, betatrophin may not be an independent marker, but mainly in correlation with C-peptide it could provide information about endogenous insulin production. We believe that further studies involving a larger, more homogeneous population of children with type 1 diabetes are necessary to clarify the exact role of betatrophin as a biomarker of pancreatic secretion.

## Data Availability

The raw data supporting the conclusions of this article will be made available by the authors, without undue reservation.
